# Structural biology inside multicellular specimens using electron cryotomography

**DOI:** 10.1017/S0033583525000010

**Published:** 2025-01-13

**Authors:** Ido Caspy, Zhexin Wang, Tanmay A.M. Bharat

**Affiliations:** 1 Structural Studies Division, MRC Laboratory of Molecular Biology, Cambridge CB2 0QH, United Kingdom

## Abstract

The electron cryomicroscopy (cryo-EM) resolution revolution has shifted structural biology into a new era, enabling the routine structure determination of macromolecular complexes at an unprecedented rate. Building on this, electron cryotomography (cryo-ET) offers the potential to visualise the native three-dimensional organisation of biological specimens, from cells to tissues and even entire organisms.

Despite this huge potential, the study of tissue-like multicellular specimens via cryo-ET still presents numerous challenges, wherein many steps in the workflow are being developed or in urgent need of improvement. In this review, we outline the latest techniques currently utilised for *in situ* imaging of multicellular specimens, while clearly enumerating their associated limitations. We consider every step in typical workflows employed by various laboratories, including sample preparation, data collection and image analysis, to highlight recent progress and showcase prominent success stories.

By considering the entire structural biology workflow for multicellular specimens, we identify which future exciting developments in hardware and software could enable comprehensive *in situ* structural biology investigations, bringing forth a new age of discovery in molecular structural and cell biology.

## Introduction

The electron cryomicroscopy (cryo-EM) resolution revolution launched structural biology into a time of unprecedented discovery, making it possible to routinely solve structures of purified macromolecular complexes at an astonishing rate (Henderson, 2004; Kühlbrandt, 2014; McCafferty et al., 2024; Nogales & Scheres, 2015). This quantum leap has set the stage for another advance where structural biology questions could be posed directly within the native, three-dimensional (3D) environment of biological specimens that range from organelles to single cells, up to tissues and whole organisms using electron cryotomography (cryo-ET) (Baumeister et al., 1999; Frank, 1995; McCafferty et al., 2024; Nogales & Mahamid, 2024). Using cryo-ET, the intricate cellular environment can now be visualised at the nanometre scale (Beck & Baumeister, 2016; Gan & Jensen, 2012).

In cryo-ET, a series of two-dimensional (2D) images of a vitrified biological sample is acquired at various tilt angles, termed tilt-series. Images in such tilt series are subsequently aligned and computationally combined to produce a three-dimensional (3D) reconstruction of the specimen, which is called a tomogram (Baumeister, 2005; De Rosier & Klug, 1968; Hoppe, 1970, 1974). Each tomogram holds within it a veritable treasure-trove of data, containing information about the molecular composition of the specimen along with its ultrastructural arrangement (Melia & Bharat, 2018; Singh et al., 2024; Xue et al., 2022; P. Zhang, 2019; Zimmerli et al., 2021).

While cryo-ET has been applied to a wide variety of specimens, there are still several difficulties associated with investigating multicellular specimens with this technique. These difficulties are specifically related to vitrification of thick specimens, sample thinning, as well as subsequent challenges in cryo-ET data acquisition and image processing. These difficulties will be addressed in turn in this article, along with some recent success stories and a balanced reflection on the future applicability of cryo-ET for near-native imaging of tissues.

## Sample preparation methods

A requirement to visualise biological specimens using cryo-ET (or cryo-EM) is that the specimen must be vitrified, meaning that the aqueous environment of the specimen of interest should form an amorphous, glass-like arrangement (Dubochet & McDowall, 1981; McDowall et al., 1983). Vitrification preserves the sample in a near-native state, providing ideal conditions for imaging while minimising radiation damage, with no artefacts in imaging caused by crystalline ice, by avoiding electron diffraction from ice crystals that corrupt the acquired data (Dubochet et al., 1988; Henderson, 1992). It is worth mentioning that while vitrification has been deemed as a necessity for cryo-EM, recent work has demonstrated reduction in beam induced motion and better reconstructions from the initial frames of the movie acquisition in a specimen devitrified in a controlled manner (Wieferig et al., 2021). Nonetheless, to prepare a suitably vitrified sample, the specimen must be cooled at a rate faster than the rate of crystalline ice formation (Dubochet et al., 1988). While most biological specimens are present in an aqueous solution, the peculiarities of each individual specimen being studied in any particular experiment determines how, practically, vitrification is performed to ensure suitable preservation for cryo-ET. Broadly, there are two major techniques that can be utilised to produce a vitrified sample – plunge freezing for thin specimens, up to ˜10 μm in thickness (Fuest et al., 2019), and high-pressure freezing for thicker specimens. For both plunge frozen and high-pressure frozen samples, the quality of vitrification must be investigated experimentally (for instance, by using electron diffraction), as this cannot be reliably assumed *a-priori*, because vitrification intimately depends on the chemical characteristics of the sample.

### Vitrification of thin samples

The conventional method of specimen vitrification for single-particle cryo-EM is to plunge the specimen into a cryogen such as liquid ethane (Bock & Grubmüller, 2022; Dubochet et al., 1988). Liquid ethane at -180 °C can generate a cooling rate of 10^6^ °C/s (Dubochet et al., 1988), thereby allowing a layer of water, generally thinner than 500 nm, to be rapidly vitrified in less than 100 μs, before the volume of the water can expand and crystalline ice of any form can manifest itself. During plunge freezing, the biological sample is applied onto a cryo-EM grid (Schaffer et al., 2017), followed by wicking the excess liquid off, to leave a thin film of the specimen on the grid, which is then plunged into the cryogen. Alternatively, cells may be grown directly on cryo-EM grids, often after the grid is coated with polymers such as poly-L-lysine or fibronectin that aid cellular adherence to the grid surface (Lam & Villa, 2021; Mahamid et al., 2016; F. R. Wagner et al., 2020). Due to the high heat capacity of the cryogen, the sample is cooled at a rapid rate, leading to efficient vitrification (Dubochet & McDowall, 1981). Additionally, samples can also be vitrified using a cryogen stream (Ravelli et al., 2020), dispensed onto a grid in minute volumes and at rapid intervals designed principally for time sensitive specimens (Dandey et al., 2020), or cryofixed during light-microscope imaging using a correlative light and electron microscope (CLEM) fitted with a microfluidics device (Fuest et al., 2019). Even more excitingly, protein samples can be passed through a mass spectrometer in a gaseous state and deposited on a cryo-cooled grid for cryo-EM, allowing an accurate characterisation of the applied specimen prior to imaging (Esser et al., 2024). These approaches offer a lot of flexibility in the sample preparation of biological material. However, for *in situ* imaging of cells and tissues, all these approaches discussed thus far are limited to relatively thin specimens, because the cooling rate drastically drops at locations away from the surface of the specimen. The thickness limitation for freezing at ambient atmospheric pressure is around 10 μm, although it varies between different biological specimens and can be slightly circumvented by the addition of cryoprotectants (Bäuerlein et al., 2023; Berger, Premaraj, et al., 2023; Fuest et al., 2019; Jentoft et al., 2023; F. R. Wagner et al., 2020).

### Vitrification of thick samples

An alternative to the approaches listed above for thin specimens is available, termed high-pressure freezing (HPF), which was developed several decades ago (Moor & Riehle, 1968), and is particularly suitable for thicker samples up to ˜200 μm (Kelley et al., 2022; Studer et al., 2008). During HPF, a pressure of ˜2100 bar is applied to biological specimens clasped between two metal planchettes during freezing. As ice is less dense than water, the high pressure hinders crystalline ice formation, thereby reducing the cooling rate requirement for vitrification (Moor, 1987). To further prevent crystalline ice from forming and improve vitrification, a cryoprotectant can be added to the sample such as glycerol (Dahl & Staehelin, 1989), glycans ( I. Y. Chang et al., 2021), polyvinyl compounds (Weil et al., 2019) and 1-hexadecene (McDonald et al., 2010). These cryoprotectants prevent the formation of crystalline ice by increasing the global concentration of all solutes present in the aqueous sample (Pegg, 2007). Another special cryoprotectant is 2-methylpentane, which can be sublimed from the vitrified specimen by heating to -150 °C, allowing additional advantages such as post-addition of fiducials, as well as for exposing the surface topography of specimens to reduce the volume that needs to be removed in the downstream thinning step (Harapin et al., 2015; S. Wang et al., 2023).

**Figure 1:**
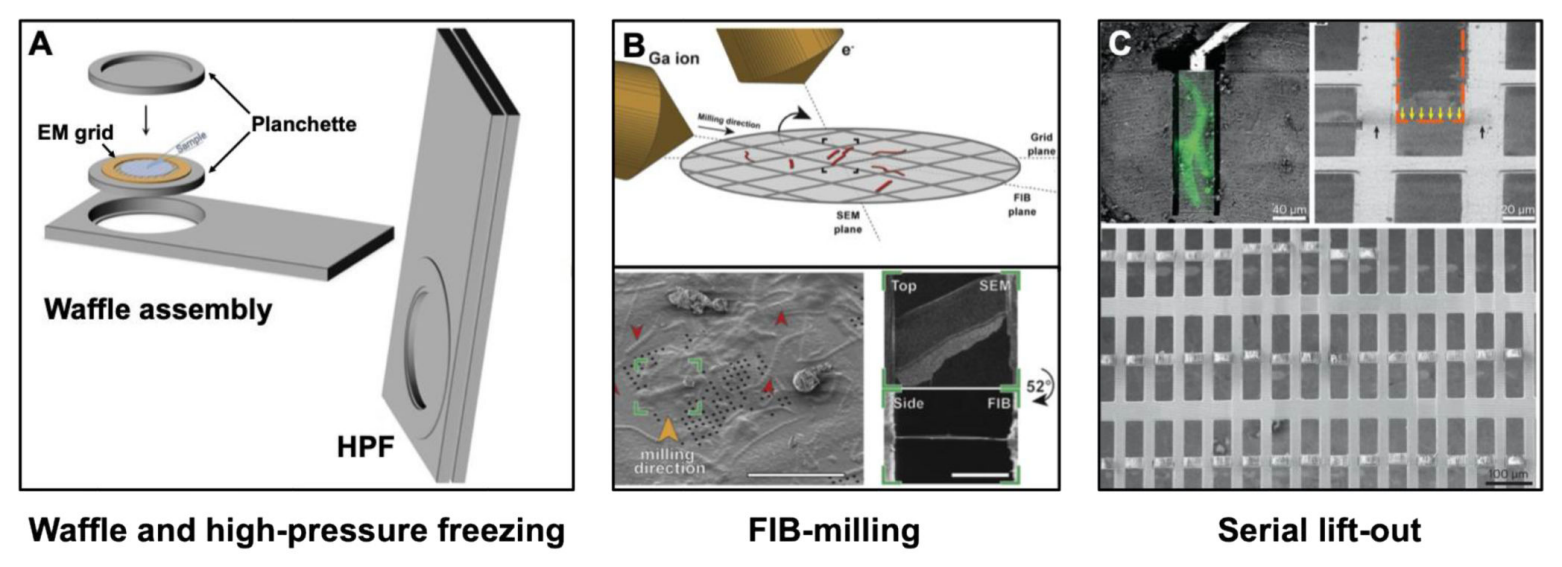
Overview of sample preparation by high-pressure freezing and FIB milling of cellular and tissue specimens. (A) Cartoon description of the waffle assembly – the EM grid is placed between two planchettes and subsequently vitrified using high-pressure freezing. Adapted from (Kelley et al., 2022). Image is CC BY, license link (http://creativecommons.org/licenses/by/4.0/). (B) Schematic showing the geometry of the focused ion beam, SEM and the grid containing the sample (top). SEM image of a plunge-frozen sample with the milling direction marked, and myofibrils are marked with red arrows (bottom left). Polished lamella images, top-view imaged with the SEM, and side-view imaged with the FIB (bottom right). Adapted from (Z. Wang et al., 2021). (C) Serial lift-out workflow: After the region of interest was identified using fluorescent labelling (green), the micromanipulator was mounted, and the area was milled in preparation for lift-out (top left). The slab removed in the previous step is positioned for subsequent thinning (top right). Overview of the milled sections prior to cryo-ET data collection (bottom). Adapted from (Schiøtz et al., 2023). Image is CC BY, license link (http://creativecommons.org/licenses/by/4.0/).

Another route to accessing thicker volumes, is to use the so-called waffle method (Figure 1A) following earlier reports of a similar nature (Weiner et al., 2013), where a grid is sandwiched between two planchettes and high-pressure frozen using the grid bars as a spacer (Kelley et al., 2022). This approach is designed to accommodate various samples at a thickness compatible with maximal reasonable gallium milling depth, which is ˜50 μm (Schaffer et al., 2019). This approach is applicable to cellular or multicellular samples, sometimes made possible by concentrating the cells (by skipping blotting), thus circumventing preferred orientation of the cells, and could be useful for purified particles as well (Kelley et al., 2022).

Yet even HPF is limited by the sample thickness and is typically useful only up to 100-200 μm (Kelley et al., 2022; Studer et al., 2008). Accessing thick tissues is currently made possible by initial mechanical sectioning using a vibratome prior to vitrification. Typically, the sample is immersed in buffer or embedded in agar, then sliced using a blade and placed on a grid for HPF (Creekmore et al., 2024; Matsui et al., 2024; J. Zhang et al., 2021). However, this step prolongs the period between sample isolation and freezing and can introduce cutting artefacts at the surface of the sample, which could hinder the preservation of the native, physiologically relevant state of interest. Thus, at the sample preparation stage, there is an urgent need to develop novel techniques for obtaining vitrified samples of much larger volumes, in particular when aiming to image entire tissues and organisms (Baumeister, 2005; Hutchings & Zanetti, 2018).

## Thinning procedures

For cryo-ET data acquisition, since the electrons must be transmitted through the biological specimen, to be able to contribute to image formation at the detector, the mean free path of electrons, or removal of inelastically scattered electrons by an energy filter, limits specimen thickness usable for cryo-ET. This thickness limit has been estimated by different studies that reported slightly different values, with some studies reporting this limit to be as low as 300 nm, because the effective thickness of the specimen increases significantly at high tilt angles during cryo-ET data acquisition, and the signal-to-noise ratio is thus greatly diminished (Petrov et al., 2022). Even for a 200 nm-thick specimen, it has been reported that the total transmitted electrons are roughly half of the illuminated dose, as they interact with the sample resulting in decoherence and energy loss (Elbaum, 2018). As most cellular specimens, apart from a few examples of smaller microbial cells (O’Reilly et al., 2020; von Kügelgen et al., 2024), are usually thicker than 200-300 nm (Melia & Bharat, 2018; Oikonomou et al., 2016), they must be thinned before cryo-ET can be performed. Previously, sample thinning for cryo-EM was only possible using cryo-ultramicrotomy, where a diamond knife is used to produce thin sections of the specimen at cryogenic temperatures. These sections are subsequently placed on an EM grid for imaging (Al-Amoudi et al., 2004; Bharat et al., 2018; Eltsov et al., 2018; Gilbert et al., 2024; Leistner et al., 2023; McDowall et al., 1983). This method, termed cryo-electron microscopy of vitrified sections (CEMOVIS), might lead to distortions in the specimen including expansion and compression due to the mechanical action of the knife. Even though CEMOVIS can be used successfully to study cellular and tissue samples *in situ* (Bharat et al., 2018; Gilbert et al., 2024; Ma et al., 2022), practical application of CEMOVIS tends to be quite tedious as the sample is prepared manually with low throughput, with the skill of the experimentalist critically determining the outcome of the procedure (Al-Amoudi et al., 2005).

### Thinning of thin(ner) specimens using ion beams

To bypass this limitation of CEMOVIS, focused-ion-beam milling (FIB milling) was adapted from material sciences and applied to biological specimens at cryogenic temperatures to obtain thin samples amenable for cryo-ET with minimal artefacts (Marko et al., 2006, 2007; Rigort, Bäuerlein, et al., 2012; Rigort, Villa, et al., 2012). For a comprehensive overview of the technique, we recommend other authoritative reviews (Noble & de Marco, 2024; Rigort & Plitzko, 2015). In brief, a focused ion beam, such as one containing gallium metal ions, is utilised to ablate biological material and mill it down to a lamella with a final thickness of roughly 180 - 200 nm (Villa et al., 2013). Before milling, the vitrified specimen is typically coated with a layer of organometallic platinum compound to protect the sample surface and to ensure that the milling process results in a smooth lamella (Schaffer et al., 2017). During milling, high gallium currents (500-1000 pA) are initially used to remove bulk materials and expose the central segment of the specimen containing the region of interest. As high currents can introduce damage to the specimen, in subsequent steps, the ion current is progressively reduced and the sample is gradually milled and polished, resulting in a thin, uniform lamella that is amenable for cryo-ET (Figure 1B) (Rigort, Bäuerlein, et al., 2012; Schaffer et al., 2017; F. R. Wagner et al., 2020). Recent studies aimed at characterising the extent of the radiation damage introduced to lamellae by the ion beam estimated that the specimen up to 30-60 nm in depth from the lamella surface is affected by milling with gallium ions (Lucas & Grigorieff, 2023; Tuijtel et al., 2024). Moreover, the data showed that lamellae thinner than 180 nm do not offer any significant improvement in the resolution obtained after subtomogram averaging, likely due to the radiation damage (Tuijtel et al., 2024). This is especially noteworthy since many groups aim for lamella thickness of 100-120 nm. In comparison for cryo-EM SPA, the ideal ice thickness has been proposed to be as small as 30 nm (Koeck & Karshikoff, 2015), although 3 Å resolution could be achieved with ice as thick as 200 nm (Neselu et al., 2023).

### Thinning of thick specimens using FIB milling

While FIB milling using gallium beams is widespread, allowing the precise generation of lamellae. The drawback is that gallium thinning is a relatively slow process, since high currents are not achievable with the available hardware configurations of the liquid metal ion sources (Burnett et al., 2016a). As a result, milling specimens thicker than 50 μm is challenging. The solution is to employ different milling strategies, use more powerful beams, or a combination of both, which will be discussed in this section. To access regions far from the tissue surface for structural studies using cryo-ET, a method termed lift-out has been developed (Mahamid et al., 2015; Rubino et al., 2012; Schaffer et al., 2019). Classical FIB-milling requires the removal of most of the material around the region-of-interest. Lift-out employs a micromanipulator with a needle or gripper at its tip to lift-out a slab that is cut off the specimen by FIB, thereby reducing the amount of material needed to be removed, in order to access deep regions. This lift-out technique is becoming more widely applied, as it allows detailed inspection of complex 3D tissue or even whole organisms in their native context. Recently, a serialised lift-out approach which produces multiple lamellae within one lift-out process has been developed to improve the throughput (Figure 1C) (Gilbert et al., 2024; Klumpe et al., 2022, 2023; Kuba et al., 2021; Nguyen et al., 2024; Plitzko et al., 2022; Schiøtz et al., 2023; Zens et al., 2024).

An alternative to using a gallium ion beam is the use of various gaseous ions produced from plasma (Berger, Dumoux, et al., 2023; Zhong et al., 2021). Plasma sources can deliver higher currents (Burnett et al., 2016a; Gorelick & Marco, 2018; Lai et al., 2017), albeit with reduced precision, which permit milling of larger volumes when compared to liquid metal sources (Berger, Dumoux, et al., 2023; Burnett et al., 2016b; Y. Chang et al., 2019; Dumoux et al., 2023; Eder et al., 2021; Parkhurst et al., 2023). Thorough examination and analysis are still required to elucidate the relative advantages of using plasma sources over liquid metal ion sources, and which gases are optimal for use in the thinning and polishing steps of lamellae preparation. Current data suggests beams using xenon plasma sources can dispose of material at a faster rate than gallium beams, suggesting that they could be useful during the rough milling step of large volumes, while argon produces lamellae at a high success rate with relatively lower radiation damage (Berger, Dumoux, et al., 2023; Berger et al., 2024; Burnett et al., 2016b). When the specimen is too thick to be thinned using FIB-milling (in the case of large organs or organisms), performing a mechanical thinning step using a vibratome and/or and an ultramicrotome presents an alternative approach to obtain a sample amenable for downstream milling (Creekmore et al., 2024; Iulianella, 2017; Matsui et al., 2024; McCafferty et al., 2024; S. Wang et al., 2023; J. Zhang et al., 2021). In the future ideally, larger areas of vitrified grids would be thinned using ion beams, making entire tissues and organisms amenable for cryo-ET data acquisition.

Various approaches in the field are currently aimed at turning cryo-FIB milling into a fully automated process, rescinding the need for high user proficiency, thus making it possible to generate more than ~50 lamellae in a single session. Hardware improvements such as the installation of cryo-shields, obtaining better chamber vacuum systems and attempts to integrate the FIB platform with TEMs to reduce contamination, all together improve the stability of the lamellae produced and increase the throughput of sample preparation for cryo-ET (Berger, Premaraj, et al., 2023; Cleeve et al., 2023; Klumpe et al., 2021; Medeiros et al., 2018; Tacke et al., 2021; Zachs et al., 2020). Future EM setups will likely include all modules present in the same type of sample holder compatible with cryo-FIB-SEM and TEM with the data collection software keeping track of the grid locations throughout the process. This will go a long way to making sample preparation and data collection more efficient, less prone to human error and with reduced contamination. Some modified setups are already available, such as the additional accessory fluorescent light-microscopes (J. Yang et al., 2023) and future setups may include mass spectrometers that could assist in localised targeting and on-the-fly compositional analysis of the specimen (Esser et al., 2024; Lindell et al., 2024; Passarelli et al., 2017).

## Cryo-ET of thinned specimens

Once the multicellular specimen has been appropriately thinned, it is ready for cryo-ET data collection for structural and cell biology. One of the major factors limiting the throughput of cryo-ET is the long acquisition time of tilt-series, compared to cryo-EM single particle analysis (Böhning & Bharat, 2021). Different data collection schemes have sought to overcome this hurdle to support widespread application of cryo-ET. One such approach accelerates the speed of a single tilt-series acquisition by implementing a continuous data collection (Chreifi et al., 2019, 2021; Eisenstein et al., 2019). In this scheme, the specimen is exposed and tilted continuously (in a single movement) without the need to track and correct stage shifts, required in standard cryo-ET data collection (Mastronarde, 2005). Abandoning these constant adjustments, which require slow mechanical stage movements in the microscope, increases the speed of tilt-series acquisition up to an order of magnitude, but limits the overall quality of the reconstructed tomograms since the tilt angle of each image must be estimated experimentally (Chreifi et al., 2019). Other approaches aimed at optimally imaging each square nanometre of the valuable milled area of the specimen include the use of overlapping tiles that are stitched together, thereby forming mosaic images that can eventually be merged and reconstructed as a highly detailed, large tomographic volume (Peck et al., 2022). Alternatively, the beam shape could be changed to a square to maximise the collection area within the lamella and permit data acquisition of neighboring areas without losing high-resolution features due to overlapping, unnecessary exposure during data collection (Brown et al., 2024; Chua et al., 2024).

**Figure 2:**
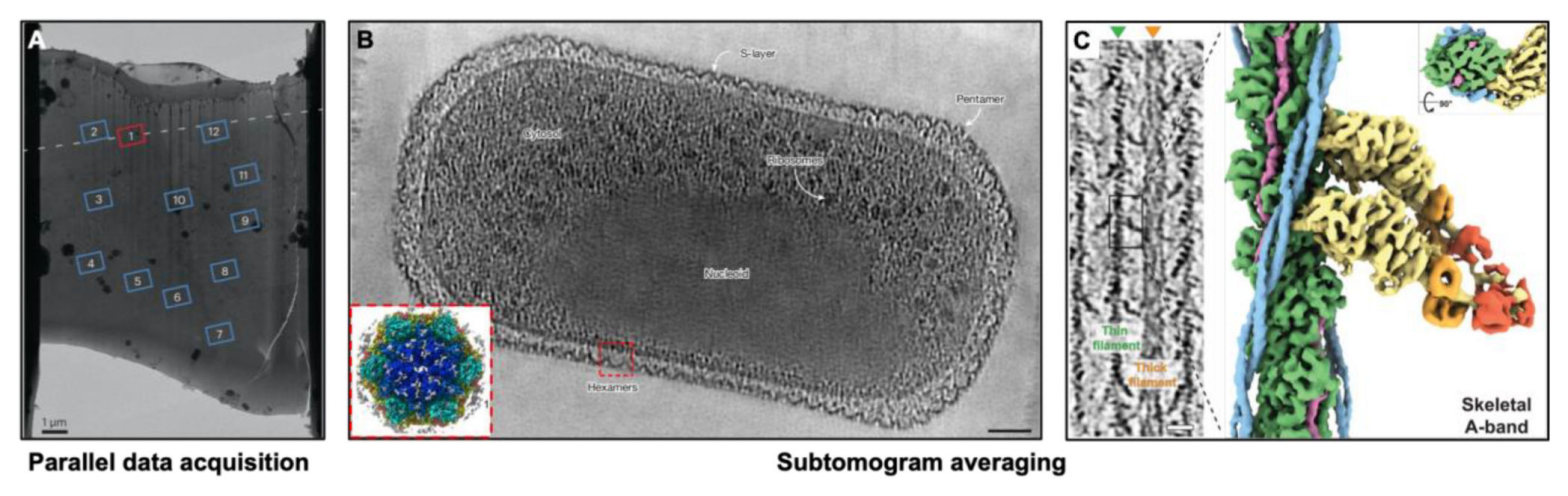
Cryo-ET data collection and high resolution subtomogram averaged structures. (A) FIB-milled lamella with defined regions for parallel cryo-ET data acquisition using beam image shifts. The tilt axis is marked with a dashed line. Adapted from (Eisenstein et al., 2022). Reproduced with permission from SNCSC. (B) Slice through a tomogram of an entire microbial cell where ribosomes, nucleoid and the surface layer (S-layer) encapsulating the cell are all visible. Inset - the subtomogram averaged map of the S-layer, adapted from (von Kügelgen et al., 2024). Image is CC BY, license link (http://creativecommons.org/licenses/by/4.0/). (C) Slice through a tomogram of the sarcomere thin and thick filaments along with the subtomogram averaged map of the thin filament with a bound myosin. Adapted from (Z. Wang et al., 2022). Reprinted with permission from AAAS.

Perhaps the most widely applicable acquisition strategy parallelises cryo-ET data collection by defining a geometric model describing the lamella surface (or any specimen surface) relative to the tilt axis. This geometric model helps in parallelised data collection by utilising beam image shifts combined with a single tracking area, hence allowing multiple tilt-series acquisition in a nearly simultaneous manner. This facilitates the collection of hundreds of tilt series in a single session, substantially increasing throughput compared to the traditional collection schemes (Bouvette et al., 2021; Eisenstein et al., 2022; J. E. Yang et al., 2023). To overcome errors introduced either by misaligned lamellae, specimen rotation caused by the mechanical autoloader system, and inaccurate measurement of the lamella’s eucentric position, the geometric model is used to compensate for these errors and is updated throughout sample tilting in the PACE-tomo (parallel cryo electron tomography) workflow (Figure 2A) (Eisenstein et al., 2022). To complement this approach and introduce further automation, a machine learning model dubbed SPACE-tomo (smart parallel automated cryo electron tomography) was trained to facilitate unattended lamella definition, identification and segmentation of regions of interest within the lamella, and data acquisition set-up (Eisenstein et al., 2023). As these methods become more widespread, we expect that such unsupervised approaches will become an indispensable part of the cryo-ET data collection pipelines.

While cryo-ET data collection has advanced significantly since the advent of direct electron detectors, there is still room to substantially improve the quality of cryo-ET data, which will potentially have a huge impact on reducing the amount of data needed to solve structures inside cells and tissues. Lamella preparation requires a lot of time and effort, therefore it is imperative that the cryo-ET data collected is of the highest possible quality (Ochner & Bharat, 2023). From a hardware standpoint, the laser phase plate is drawing significant attention and holds the potential to substantially improve the signal-to-noise ratio in cryo-ET by modulating the phase contrast difference between scattered and unscattered electrons, during in-focus specimen imaging (Müller et al., 2010). The unscattered electrons are focused and passed through an electric field generated by an ultrafast continuous (Schwartz et al., 2019) or pulsed (Du & Fitzpatrick, 2023) laser. The passage through the field induces a phase shift caused by the ponderomotive force, resulting in a phase difference (π/2 in the case for a quarter phase plate), thus boosting contrast significantly even when the specimen is in focus (Du & Fitzpatrick, 2023; Müller et al., 2010; Schwartz et al., 2019). The phase contrast of the detected electrons results in better image quality by converting the sine oscillation to cosine, thus increasing the low-frequency signal, although CTF correction is still required for both low and high resolution (Campbell et al., 2018; Müller et al., 2010; Petrov et al., 2022; Schwartz et al., 2019). The drawback of the current design was described as a resolution loss due to magnetic field fluctuations caused by the laser pulses, which are currently being investigated for future improvements (Axelrod, Petrov, Zhang, Remis, et al., 2023; Axelrod, Petrov, Zhang, Sandhaus, et al., 2023).

## Subtomogram averaging structure determination

Once cryo-ET data on the multicellular specimen has been collected, subtomogram averaging (STA) can be applied to obtain structural information from the macromolecules present within the specimen. Subtomogram averaging approaches have been described in other reviews focused on this method (Briggs, 2013; Lučić et al., 2013), therefore it is only considered here briefly for completeness. Following tilt-series acquisition, tomograms can be reconstructed in a variety of software (Kremer et al., 1996; Zheng et al., 2022) using a variety of pipelines (Burt et al., 2024; Himes & Zhang, 2018; H. F. Liu et al., 2023). Next, typically tomographic volumes are denoised to improve contrast (Buchholz et al., 2018; Y. T. Liu et al., 2022), after which researchers can use a variety of tools for manual picking, template matching or other feature identification tasks (Chaillet et al., 2024; Cruz-León et al., 2024; de Teresa-Trueba et al., 2023; Lucas et al., 2023; Moebel et al., 2021; Rice et al., 2023; T. Wagner et al., 2019, 2020; Wan et al., 2024). These subtomogram selection tasks can be followed by classification and subtomogram averaging (Burt et al., 2024; M. Chen et al., 2019; Förster et al., 2005; H. F. Liu et al., 2023; Tegunov et al., 2021). Subtomogram averaging allows structure determination of macromolecules in their native environment (Figure 2B-C) (Allegretti et al., 2020; S. Chen et al., 2024; Z. Chen et al., 2023; Fedry et al., 2024; Gemmer et al., 2023; Q. Guo et al., 2018; Held et al., 2024; Hoffmann et al., 2022; Hutchings et al., 2018; Kravčenko et al., 2024; Leung et al., 2023; Mattei et al., 2016; Ni et al., 2022; Obr et al., 2024; Pyle et al., 2024; Santos et al., 2024; Schur et al., 2016; Turoňová et al., 2020; von Kügelgen et al., 2020, 2024; J. Wagner et al., 2024; Waltz et al., 2024; Z. Wang et al., 2021, 2022; Watanabe et al., 2020, 2024; Wozny et al., 2023; Xue et al., 2022; You et al., 2023; X. Zhang et al., 2023; Zimmerli et al., 2021), using image processing algorithms that support high-resolution structure determination (Bharat et al., 2015; Tegunov et al., 2021; Zivanov et al., 2022). The resulting structures provide valuable insights on the mode of action of macromolecules in tissues, along with their interactions with drugs, ligands, or accessory molecules *in situ*. These interactions are often transient or disrupted by protein purification techniques and thus cannot be easily reconstituted and visualised *in vitro*.

Several modern studies not only report the cellular structures of macromolecules by STA, but also map the resulting structures back into the original tomogram, providing additional ultrastructural information of the tissue. With this in mind, we must note that a thinned sample is taken out of the cellular or tissue context, because once thinned, it represents only a small slice from the initial intact specimen. We anticipate that in the next few years, more molecular structures will be characterised using a workflow combining cryo-FIB-SEM, cryo-ET, predictive algorithms (Jumper et al., 2021) and cellular transcriptomics and proteomics approaches (Baumeister, 2005; McCafferty et al., 2024).

## Complementary techniques for 3D *in situ* imaging

Cryo-ET provides molecular resolution in a limited sample volume, due to the requirement of thinning tissue specimens. This limitation can be partially alleviated by montage tomography (Peck et al., 2022; J. E. Yang et al., 2023), which expands the field-of-view in the “X-Y” dimension, and by serial lift-out approaches, which increases depth through fabrication of multiple lamellae from the same tissue (Nguyen et al., 2024; Schiøtz et al., 2023). However, this loss of sample volume due to thinning is to an extent unavoidable in cryo-ET. To circumvent this issue, there are other *in situ* imaging techniques that provide an alternative option for imaging bulk volumes such as 3D FIB-SEM imaging (also termed serial surface imaging or “slice-and-view”), where a layer of biological material is removed using the FIB followed by imaging of the exposed surface using the SEM. By iterating the FIB-SEM process, a 3D volume can be generated with nearly isotropic resolution of a few nanometres. This technique is extremely useful for cell biological investigations inside tissues, because it provides a large fields-of-view, and depth information through the “Z”-axis of the tissue (Elbaum, 2018). This serial FIB-SEM technique had previously been widely applied for room temperature specimens that were chemically fixed (Denk & Horstmann, 2004; D’Imprima et al., 2023; Heymann et al., 2006; Xu et al., 2017, 2021), and has been recently expanded to cryogenic temperature applications (Capua-Shenkar et al., 2022; Scher et al., 2021; Schertel et al., 2013; Sviben et al., 2016; Vidavsky et al., 2016). Despite the large potential applications, several challenges remain in the pipeline for imaging cryogenic, unstained biological specimen, such as problems with automatic focusing, automatic astigmatism and drift correction on these radiation sensitive samples that are imaged for several hours, and sometimes several days. Moreover, interpretation of the resulting images remains complex due to the incompletely understood mechanisms of contrast formation of cryogenic, unstained biological specimens. While the contrast is suggested to arise from differential surface potential and local charging, additional factors may also contribute (Schertel et al., 2013; Vidavsky et al., 2016). With the growing attention on cryo-FIB-ET, the cryo-FIB-SEM technique is expected to become more widely accessible, as it can be performed using the same instrumentation available in many laboratories for lamella production. Widespread application will likely require theoretical developments in understanding image formation, and in the development of streamlined strategies for data analysis. We hope further software and hardware advancements will address the current challenges, for example by reducing the ion beam size to allow finer slicing of the sample, as well as improved SEM detectors that can decrease the dwell time and allow faster imaging.

In the same vein as FIB-SEM imaging, another alternative method to investigate whole cells or tissues in 3D is cryo-Scanning Transmission Electron Microscopy (STEM), which uses a focused electron beam probe rather than flood beam used in TEM applications (Jones & Leonard, 1978; KELLENBERGER et al., 1986). Cryo-STEM allows scanning over the sample in a tiled manner using multiple detectors that collect information for both transmitted and scattered electrons (Elbaum et al., 2021; Wolf & Elbaum, 2019). While samples up to 2 μm in thickness can potentially be imaged using cryo-STEM, in practice to obtain data with a good contrast and a reasonable pixel size the effective specimen thickness is usually less than 1 μm (Kirchweger et al., 2023; Wolf et al., 2014). Cryo-STEM has been successfully utilised to visualise whole cells (Wolf et al., 2014), organelles containing granular calcium structures (Kirchweger et al., 2023; Wolf et al., 2017), single particle reconstructions at sub-nanometre resolutions of purified proteins and virus particles (Lazić et al., 2022), as well as metal ion composition and localisation in purified proteins (Elad et al., 2017). Cryo-STEM is therefore a complementary technique for cellular imaging, providing another quiver in the arrow of the *in situ* structural cell biologist.

Another cryo-tomography technique which has been recently used to investigate large cells and tissues, albeit at lower resolution, is cryo-soft X-ray tomography (cryo-SXT), which can provide information through specimens that are several microns thick (Larabell & Le Gros, 2004; Weiß et al., 2000). In cryo-SXT, contrast is naturally generated by the difference in the K-shell absorption of soft X-rays between carbon (or nitrogen) and oxygen in wavelengths ranging 2.34-4.4 nm (Larabell & Nugent, 2010). Imaging in this spectral range, also termed the “water window”, causes organic material, which is abundantly present in cells and organelles to absorb the X-rays, while water and other oxygen rich compounds are effectively transparent (Carzaniga et al., 2014; Larabell & Le Gros, 2004). Cryo-SXT offers not only a large depth of field, which can reach 10-15 μm (Carzaniga et al., 2014; J. Guo & Larabell, 2019; Uchida et al., 2009), but also a large field of view together with fast data acquisition times, where unstained and unmodified cells nearly 50 μm in length can be imaged in 20 minutes with a resolution of about 50 nm (Larabell & Nugent, 2010; Uchida et al., 2009). This is much faster when compared to 3D FIB-SEM or cryo-STEM which take several hours or days to collect a dataset of a similar scale. Recent advancements in cryo-SXT include improvement of data collection schemes to increase the depth of field (Otón et al., 2017), however the most substantial is the transition from synchrotron-based microscopes into compact, standalone machines which can be operated in a typical laboratory (Fahy et al., 2021, 2024), which is expected to make this method available to a wider community.

## Conclusions and outlook

In conclusion, we have reviewed recent advances pertaining to sample preparation, thinning strategies and cryo-ET data collection schemes, which are currently being used to investigate multicellular specimens and tissues *in situ*. From the sample preparation perspective, there is currently no method that can allow a reliable vitrification of specimens thicker than 100-200 μm, meaning that most tissues are currently not directly amenable for imaging by cryo-ET, and innovation in this aspect is urgently needed. This could be achieved by repurposing HPF to accommodate thicker specimens, or by devising alternative techniques for sample preparation. While metal ion beam sources have been used extensively in materials sciences as well as for biological cryo-FIB sample thinning, they are still limited by a low rate of material removal and hence prevent easy access to thicker tissue samples. Investigating different focused ion beams is required to allow faster and more reliable milling, ideally with the potential to reduce the damage the sample undergoes during thinning. As automation and increased throughput are introduced to the FIB milling process, cryo-ET data collection must also improve to allow tomogram acquisition from multiple lamellae across multiple grids. Beam-shift collection schemes could greatly increase the rate of data collection without compromising data quality, but there is room for improvement in making this available for all sorts of applications. To tackle the densely packed cellular environment, and increase the overall contrast of tomograms, the laser phase plate is expected to push the limits of macromolecular identification in tomograms. These and other approaches might help generate higher-resolution tomograms, where sub-nanometre-level details could be resolved and inferred directly from the reconstructed tomogram, without the need for subtomogram averaging. We envision that future cryo-EM instruments will include a combination of multimodal components such as cryo-FIB-SEM, light microscopy objectives and mass spectrometers, that will complement TEM data acquisition, with cryo-ET as the central method of choice linking information from these diverse sources together to help uncover new biological mechanisms.
